# Pre-operative circulating tumour DNA in high-risk primary cutaneous melanoma: prospective feasibility in molecular pathology

**DOI:** 10.2340/1651-226X.2026.45921

**Published:** 2026-09-09

**Authors:** Magnús Pétur Bjarnason Obinah, Estrid Høgdall, Tim Svenstrup Poulsen, Karin Dreisig, Thomas Litman, Christoffer Johansen, Stig Egil Bojesen, Lisbet Rosenkrantz Hölmich

**Affiliations:** a Department of Plastic Surgery, Copenhagen University Hospital – Herlev and Gentofte, Copenhagen, Denmark; b Molecular Unit, Department of Pathology, Copenhagen University Hospital – Herlev and Gentofte, Copenhagen, Denmark; c Department of Clinical Biochemistry, Copenhagen University Hospital – Herlev and Gentofte, Copenhagen, Denmark; d Department of Immunology and Microbiology, Faculty of Health and Medical Sciences, University of Copenhagen, Copenhagen, Denmark; e Centre for Cancer Late Effect Research CASTLE, Department of Oncology, Copenhagen University Hospital – Rigshospitalet, Copenhagen, Denmark; f Department of Clinical Medicine, Faculty of Health and Medical Sciences, University of Copenhagen, Copenhagen, Denmark

**Keywords:** Cutaneous malignant melanoma, circulating tumour DNA, liquid biopsy, high-throughput nucleotide sequencing, risk assessment, polymerase chain reaction

## Abstract

**Background and purpose:**

Circulating tumour DNA (ctDNA) has emerged as a prognostic biomarker in melanoma, but its detectability in pre-operative blood from patients presenting with primary cutaneous melanoma remains incompletely evaluated. We assessed the feasibility of pre-operative ctDNA detection in high-risk primary melanoma using routinely available methods in molecular pathology.

**Patients/material and methods:**

In a prospective single-institution cohort enrolled between September 2021 and December 2022, pre-operative plasma was obtained from patients with clinically suspected primary cutaneous melanoma. Patients with pathologically confirmed invasive melanoma and high-risk features (≥ T3a, ≥ N1a, or ≥ M1a) were selected for molecular analysis. Tumour tissue was analysed using next-generation sequencing (NGS) to identify targetable *BRAF* or *NRAS* driver mutations, and pre-operative plasma was analysed for ctDNA using droplet digital PCR (ddPCR) for *BRAF* V600E or targeted NGS for other driver mutations.

**Results:**

Of the 288 consented patients with pre-operative blood samples, 21 met high-risk criteria, and 12 had a targetable *BRAF* or *NRAS* driver mutation in tumour tissue and underwent tumour-informed plasma ctDNA analysis. Pre-operative ctDNA was not detected in any of these 12 patients (0 of 12; 95% confidence interval [CI] 0 to 26.5%). All ddPCR and NGS assay controls performed as expected, and wild-type copy counts were consistent across ddPCR samples.

**Interpretation:**

The finding is concordant with two independent studies using different methods, and a mathematical prediction of ctDNA shedding from small primary tumours. Reliable pre-operative ctDNA detection in primary melanoma may require alternative cell-free DNA (cfDNA) approaches, such as bespoke multivariant or mutation-agnostic methods.

## Introduction

The incidence of cutaneous melanoma has risen steadily in fair-skinned populations [1], with recurrence rates varying substantially by stage at diagnosis – from 2% in stage IA to 71% in stage IIID across the observed follow-up period [2]. Accurate risk stratification therefore guides adjuvant treatment decisions and surveillance and currently relies on primary tumour histopathology and sentinel lymph node biopsy (SLNB). However, conventional staging can fall short when primary tumour and/or nodal status cannot be adequately assessed, for example, after curettage or when SLNB is unsuccessful, and even when staging is successfully completed, prognosis can differ substantially within stages. Tissue-based molecular tools such as the clinicopathological and gene expression profile (CP-GEP) model address this in part by combining Breslow thickness and age with tumour gene expression to identify patients at low risk of nodal metastasis [3].

Circulating tumour DNA (ctDNA), a tumour-derived subset of cell-free DNA, has emerged as a prognostic biomarker in melanoma. In stage III disease, detectable ctDNA around the time of intended curative surgery predicts shorter recurrence-free and melanoma-specific survival [4–6]. In a study on clinical stage IIIB/C melanoma, detectable ctDNA following neoadjuvant immunotherapy and subsequent surgery was 100% predictive of recurrence [7].

Few studies have measured ctDNA before excision of primary cutaneous melanoma, and the available studies differ markedly in stage composition, assay design, and clinical setting, ranging from highly specific BRAF-only assays in mixed non-metastatic cohorts [8] to bespoke multivariant tumour-informed assays in resectable high-risk disease [9, 10]. Whether ctDNA can be reliably detected pre-operatively in patients presenting with melanoma, using methods routinely available in molecular pathology, therefore remains unresolved.

We present a prospective feasibility study addressing this question using ddPCR and targeted NGS workflows available in routine molecular pathology, and thus more accessible than the bespoke assays used in prior studies.

## Patients/material and methods

### Study design and participants

This prospective single-institution study at Copenhagen University Hospital – Herlev and Gentofte investigated whether ctDNA could be detected in pre-operative blood samples from patients with primary cutaneous melanoma. Between September 2021 and December 2022, we included patients aged 18 or older, referred through the Danish cancer patient pathway with suspicion of primary melanoma, excluding those pregnant or unable to provide consent.

A pre-operative blood sample was drawn before surgical excision. Blood was collected and processed using the same protocol as a parallel ctDNA recurrence-detection study: two 9 mL EDTA tubes, centrifuged at 3,000 × g for 6 min within 3 h of sampling, with 8 mL of plasma recovered and stored at −80°C [11] (Supplementary Methods).

### Patient selection

To maximise the likelihood of ctDNA detection in this feasibility study, tissue and plasma analysis was restricted to patients meeting pre-specified high-risk criteria: T3a or higher, N1a or higher, or M1a or higher. Staging was established from the primary excision, SLNB, and whole-body ^18^F-FDG PET-CT performed in cases of thicker primary tumours (T3b or higher) or positive sentinel nodes.

### Tissue analysis

For selected patients, DNA was extracted from formalin- fixed paraffin-embedded (FFPE) primary tumour tissue. Haematoxylin-and-eosin-stained slides were reviewed by a specialised pathologist, who identified areas with a minimum tumour cell content of 20%, from which tissue cores were retrieved using 1-mm disposable punchers. DNA was analysed using the Oncomine Tumor Mutation Load Assay (Thermo Fisher Scientific, Waltham, Massachusetts, U.S.), a targeted next-generation sequencing (NGS) panel [12]. Tissue sequencing was performed at a minimum coverage of 500× and a variant allele frequency (VAF) threshold of 5%, and all called variants were evaluated individually by experienced molecular biologists.

Patients proceeded to plasma analysis if their tumour harboured a targetable *BRAF* or *NRAS* driver mutation. Patients with clinical pathology confirming a *BRAF* V600E mutation in the resected primary tumour proceeded directly to plasma analysis without additional study NGS.

The final plasma cohort was thus conditional on both high-risk pathological features and the presence of a targetable *BRAF* or *NRAS* driver mutation.

### Plasma analysis

Cell-free DNA (cfDNA) was extracted from pre-operative plasma samples. Samples from patients with a *BRAF* V600E mutation were analysed using ddPCR, while samples from patients with other detected mutations, including non-V600E *BRAF* mutations, were analysed by targeted NGS, reflecting the local availability of validated assays for identified mutations.

For ddPCR analysis, a clinically validated BRAF V600E assay was used, reported to detect variant allele fractions down to 0.5%, with a limit of blank of 0 copies and 100% sensitivity and specificity against routine diagnostic testing [13]. Extracted cfDNA was distributed across nine replicate wells (5 μL per well), and results were pooled across wells for each patient. Positive ctDNA detection was defined as a mutant copy concentration exceeding the limit of detection (LoD) of 0.6 copies/mL for 8 mL plasma samples, based on an internally established and externally quality-controlled threshold. The limit of quantification (LoQ) was 7.2 copies/mL.

For NGS analysis, ctDNA was detected using the Oncomine Tumor Mutation Load Assay. Thresholds for plasma cfDNA were established de novo for this study, as plasma NGS with this panel has not been previously reported by our laboratory; this workflow was therefore exploratory. Positive detection was defined as the presence of the tumour-specific variant at a VAF at or above an LoD of 0.1%, with variant-supporting reads required on both forward and reverse strands at a minimum coverage of 5,000×.

For full methodological details, see Supplementary Methods.

### Statistical analysis

Analyses were descriptive and conducted using R version 4.3.2 (R Foundation for Statistical Computing, Vienna, Austria). Categorical variables were summarised as counts and percentages, continuous variables as medians and full ranges. The ctDNA detection rate was reported with a 95% Clopper-Pearson exact confidence interval (CI). No inferential tests were performed, as the observed detection rate precluded meaningful subgroup comparisons. Patients were enrolled consecutively, and laboratory personnel were blinded to clinical details beyond study identification numbers. No data were missing for the variables reported or for the ctDNA outcomes.

## Results

### Cohort selection

**Figure 1:**
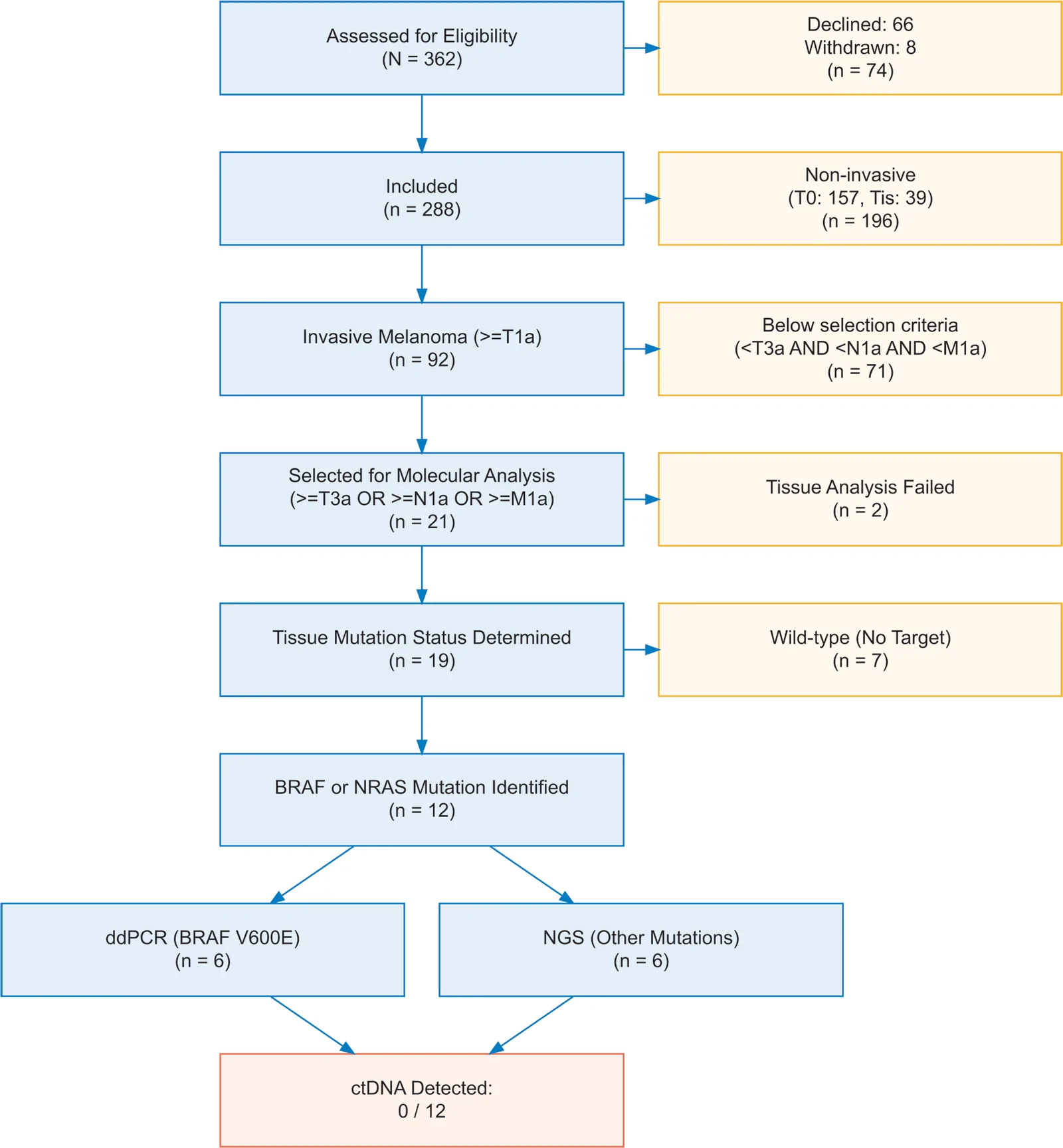
Study flowchart of patient inclusion, selection and analysis.

Of the 362 patients referred through the Danish cancer patient pathway with suspicion of primary melanoma between September 2021 and December 2022, 288 consented and underwent pre-operative blood sampling (Figure 1). Final pathological diagnoses were ascertained afterwards, confirming invasive melanoma in 92 patients, of whom 21 met the study criteria for molecular analysis.

### Tissue mutation detection

Tumour tissue NGS analysis was performed on 19/21 tumours – as two patients had a *BRAF* V600E mutation confirmed by clinical pathology during treatment – and succeeded in 17/19 samples (DNA extraction failed in one, and sequencing failed in one). A targetable *BRAF* or *NRAS* driver mutation was identified in 10/17 successfully analysed samples, while seven samples were wild-type. In total, 12/21 patients selected for molecular analysis had a targetable driver mutation and proceeded to ctDNA analysis of pre-operative plasma. Targeted mutations were *BRAF* V600E (*n* = 6, 50%), *BRAF* V600K (*n* = 2, 17%), *BRAF* K601E (*n* = 1, 8%), *NRAS* Q61K (*n* = 2, 17%), and *NRAS* Q61R (*n* = 1, 8%).

### Patients’ characteristics

Among the 12 analysed patients, the median age was 65 years (range 30 to 80), and eight (67%) were male. Primary tumours were located on the trunk (*n* = 4), upper extremity (*n* = 4), lower extremity (*n* = 3), or head and neck (*n* = 1). Histologically, eight tumours (67%) were superficial spreading melanoma and four (33%) were nodular melanoma. Five tumours (42%) were ulcerated (Table 1). Mitoses were present in 11 tumours (92%). Median Breslow thickness was 2.25 mm (range 0.8 to 10.0). Seven patients (58%) had node-positive stage-III disease, including all five with a Breslow thickness at or below 2.0 mm. According to American Joint Committee on Cancer (AJCC) 8th edition pathological staging, three patients (25%) had stage IIB, two (17%) stage IIC, five (42%) stage IIIA, and two (17%) stage IIIB disease (Table 2). The characteristics of analysed and non-analysed patients are compared in Supplementary Table 1.

**Table 1:** Demographics and clinicopathological characteristics of the 12 analysed patients.

Characteristic	Value
**Sex**	
Male	8 (67%)
Female	4 (33%)
**Age at inclusion, years: median (range)**	65 (30–80)
**Tumour location**	
Head/Neck	1 (8%)
Trunk	4 (33%)
Upper extremity	4 (33%)
Lower extremity	3 (25%)
**Histological subtype**	
Superficial spreading	8 (67%)
Nodular	4 (33%)
**Ulceration**	
Yes	5 (42%)
No	7 (58%)

**Table 2:** Per-patient tissue mutations and pre-operative plasma ctDNA results (n = 12).

Breslow (mm)	N-Stage	AJCC stage	Tissue mutation	Plasma method	ctDNA detected
2.3	N0	IIB	NRAS Q61R	NGS	Not detected
2.6	N0	IIB	BRAF V600E	ddPCR	Not detected
3.0	N0	IIB	BRAF V600K	NGS	Not detected
6.0	N0	IIC	BRAF V600E*	ddPCR	Not detected
10.0	N0	IIC	BRAF V600K	NGS	Not detected
0.8	N1a	IIIA	BRAF V600E	ddPCR	Not detected
0.8	N1a	IIIA	NRAS Q61K	NGS	Not detected
1.1	N1a	IIIA	BRAF K601E	NGS	Not detected
1.4	N1a	IIIA	NRAS Q61K	NGS	Not detected
1.6	N1a	IIIA	BRAF V600E	ddPCR	Not detected
2.2	N1a	IIIB	BRAF V600E	ddPCR	Not detected
3.0	N1a	IIIB	BRAF V600E*	ddPCR	Not detected

AJCC: American Joint Committee on Cancer; NGS: next-generation sequencing; ddPCR: droplet digital PCR; ctDNA: circulating tumour DNA.

* Mutation identified from clinical tissue mutation analysis, not study NGS.

N1a denotes a single clinically occult, microscopically detected tumour-involved node (AJCC 8th Ed.)

### Plasma ctDNA detection

Pre-operative ctDNA was not detected in any of the 12 analysed patients (0 of 12; 95% CI: 0 to 26.5%), neither using ddPCR for BRAF V600E (*n* = 6), nor NGS for other targeted driver mutations (*n* = 6). Wild-type copy counts were consistent across all ddPCR samples, and all ddPCR and NGS assay controls performed as expected.

## Discussion and conclusion

We found no detectable ctDNA in pre-operative plasma from 12 patients with high-risk primary cutaneous melanoma, using ddPCR and targeted NGS. This result can reflect both the underlying biology of ctDNA shedding and the inherent limits of current cfDNA-based detection strategies. This is the first prospective evaluation of pre-operative ctDNA detection in primary melanoma using ddPCR and targeted NGS workflows available in routine molecular pathology.

Pre-operative ctDNA detection in primary melanoma has only been examined in relatively few cohorts, and across these reports, there is a consistent pattern – detection is rare in early-stage disease with low tumour burden and increases mainly with advancing stage rather than with assay sensitivity alone. Our 0 of 12 result is consistent with Brunsgaard et al., who detected ctDNA in only one of nine (11%) patients with pathologic stage IIB/C using a 16-variant tumour-informed assay [9], and the Serial ctDNA Monitoring as a Predictive Biomarker in Advanced NeoplAsms (SAMBA) study, which detected no ctDNA at any timepoint in 12 stage IIB–IIIA patients, despite using a 48-variant tumour-informed assay [10].

Higher detection rates reported in other clinical settings do not contradict these results. Lee et al. studied a fundamentally different patient group – primary excised, predominantly macroscopic stage III nodal disease still *in situ* before complete lymph node dissection – and reported a 33% detection rate, albeit exclusively in patients with nodal deposits ≥10 mm [4]. Similarly, Chan et al. reported ctDNA detection in 48% of patients with measurable stage IIIB/C disease before neoadjuvant immunotherapy [7]. Gouda et al. reported a 36.8% pre-operative detection rate, using pre-amplified ultrasensitive BRAF V600E ddPCR in a mixed stage 0-III cohort, though with substantial plasma-tissue discordance [8].

Three cohorts of early-stage primary melanoma – ours (0/12), Brunsgaard (1/9), and SAMBA (0/12) – using assays tracking 1, 16, and 48 mutations, respectively, all produced comparably low detection. That the substantially larger bespoke panels used by Brunsgaard and SAMBA did not meaningfully improve detection suggests that within this range, the limit is the amount of ctDNA shed into plasma, rather than assay sensitivity or the number of variants tracked. As these cohorts differ in stage composition, plasma volume, assay design and sampling timing, this comparison is indirect and hypothesis-generating rather than confirmatory.

The underlying biology supports this interpretation. Applying the Avanzini biophysical model of ctDNA shedding [14] to our cohort’s tumour profile (median Breslow 2.25 mm; eight of 12 tumours less than 3 mm; nodal metastases limited to N1a microscopic) predicts a tumour fraction of approximately 0.003%, corresponding to less than one mutant copy per 8 mL plasma. This is below the LoD of both ddPCR (0.6 copies/mL) and the Oncomine plasma NGS panel (Supplementary Methods).

The strengths of this study include the prospective design, consecutive enrolment, successful tissue mutation profiling in 17 of 19 selected patients, and the use of two analytically distinct methods. Several limitations apply. The *n* = 12 cohort is small, with a 95% CI extending to 26.5%. Plasma NGS QC metrics were not retained, precluding retrospective confirmation of sequencing depth. The Avanzini framework is calibrated on non-small-cell lung cancer, and its applicability to melanoma has not been empirically validated. Selection based on the presence of a targetable BRAF or NRAS mutation may also have introduced a biological bias of unknown significance. Finally, these findings apply to conventional ddPCR and targeted NGS in routine molecular pathology and do not generalise to bespoke multivariant or mutation-agnostic approaches.

In conclusion, pre-operative ctDNA from primary cutaneous melanoma was not detectable in this cohort using standard ddPCR and targeted NGS. The clinical rationale for pre-operative molecular staging persists where conventional methods fail, but these findings, together with prior negative bespoke-assay studies, suggest that increasing the number of tracked variants alone, within the range examined, may be insufficient to overcome the biological limits of ctDNA shedding in primary melanoma. Bespoke ultra-sensitive cfDNA assays, reported to reach a LoD of a few parts per million [15], and mutation-agnostic approaches, such as fragmentomics [16] and methylation-based detection [17], may warrant evaluation in this setting. These are exploratory feasibility observations in a small cohort and should not be read as evidence against the clinical utility of pre-operative ctDNA assessment in melanoma.

## Supplementary Material



## Data Availability

Due to patient confidentiality requirements, access to the complete dataset will be restricted to active clinical members of the study group. Qualified researchers may request access to data through the corresponding author, subject to appropriate data sharing agreements and ethical approvals.
